# Climate, Carbon Dioxide, and Plant-Based Aero-Allergens: A Deeper Botanical Perspective

**DOI:** 10.3389/falgy.2021.714724

**Published:** 2021-08-20

**Authors:** Lewis H. Ziska

**Affiliations:** Associate Professor, Mailman School of Public Health, Columbia University, New York, NY, United States

**Keywords:** allergic rhinitis, asthma, aeroallergens, carbon dioxide, climate change, ozone, volatile organic carbons

## Abstract

There is global evidence of a general increase in the incidence and prevalence of respiratory diseases including allergic rhinitis and associated asthma. This increase in turn, has been related, in part, to concurrent increases in carbon dioxide (CO_2_) and temperature on pollen production and allergic disease generated from plant-based sources of pollen. Such links to anthropogenic climate change has suggested three significant and interrelated consequences associated with respiratory allergies or disease. First, warmer temperatures and a longer frost-free growing season can influence pollen season length and temporal exposure to airborne aeroallergens. Second, both warmer temperatures and additional CO_2_ can increase the amount of pollen, the seasonal intensity, from spring through fall. Thirdly, there is evidence from oak and ragweed that rising levels of CO_2_ could increase the allergen concentration of the pollen and symptom severity. However, while these outcomes are of obvious consequence, they do not fully encompass all of the plant derived changes that could, directly or indirectly, influence aeroallergen production, exposure, and consequences for public health. In this overview, I will delve deeper into other plant-based links to climate/CO_2_ that are consequential either directly or indirectly to allergic rhinitis and associated disease. Such interactions range from pollen morphology to fire occurrence, from volatile organic compounds to potential changes in pesticide usage. The goal in doing so is to provide a broader context and appreciation for the interactions between plant biology and climate that can also affect allergen production and human impact but which, to date, have received little recognition or research.

## Introduction

There is wide-spread documentation that allergenic diseases have risen significantly in the last half century (1). Clinical evidence indicates increases in the incidence and prevalence of allergenic diseases including allergic rhinitis and asthma (2, 3).

While the basis for the upturn in respiratory diseases is multi-factorial (e.g., air pollution, lifestyle), there is substantial evidence that plant sources of allergenic pollen are also increasing in response to climatic change (4). An in-depth historical analysis of pollen records across the northern hemisphere has indicated that the ongoing increase in temperature extremes (Tmin and Tmax) might already be contributing to extended seasonal duration and increased pollen load for multiple aero-allergenic pollen taxa (5). A recent report (6) used a range of models and found that climate change was the dominant driver of changes in pollen season length and a significant contributor to increasing pollen concentrations in North America. In addition, there is also evidence that recent and projected levels of CO_2_, the primary source of carbon for plant photosynthesis, can also increase allergenicity of ragweed and oak pollen (7, 8).

These three aspects, pollen load, seasonality, and allergenicity are of obvious importance. Yet, they do not represent the full spectrum of interactions between plant biology, CO_2_ and climate with respect to aero-allergen production, distribution, and impact. Those interactions, while not as recognized, will also have subsequent effects specific to allergic disease. The goal of the current review is to elucidate those direct or indirect consequences; provide, when possible, a biological basis for their potential interactions, and to offer guidance for future research directions and unmet needs. In this review, direct, relates specifically to aspects of plant biology including development, morphology, and ecology; indirect, refers to interactions with other biotic or abiotic shifts likely to occur in conjunction with climate change.

## Direct Effects

### Height and Wind Speed

Climate and rising levels of CO_2_, in addition to altering pollen production, can also influence the release and dispersion of pollen. Plant height can be a factor in distance traveled for pollen in anemophilous plants, but also for seed—influencing the rate of spread of known aero-allergenic plants such as ragweed (9, 10). Wind patterns and wind intensification are factors expected to respond to climate change (11, 12). For example, Zeng et al. (13) reported that winds across North America, Europe and Asia have been intensifying since about 2010, with the global average wind speed increasing. Such an increase will have subsequent consequences for distance and longevity of pollen within air currents. Recently, Menzel et al. (14) showed that pollen transport from non-native sources could be a factor in the length, timing and severity of the allergenic pollen season. However, climate induced changes in wind patterns related to temporal and quantitative changes in pollen remain largely uncategorized.

### Pollen Morphology, Fragmentation, Allergenicity

Warmer temperatures during anthesis can result in morphological and anatomical changes in pollen development, often leading to pollen sterility (15, 16). Temperature may also have implications as to chemical composition and structural deformity of pollen [e.g., peas, (17)]. However, these structural changes have not been extensively evaluated with respect to key pollen characteristics associated with allergenicity, such as structural integrity or changes in allergenic protein concentration.

Temperature (or CO_2_) induced changes in pollen structural integrity may of particular importance during heavy rainfall which can induce pollen rupturing into submicrometer pollen fragments (18). These fragments can penetrate deeper into human lungs and can persist for longer periods in the atmosphere. Such fragments can peak during convective thunderstorms, thunderstorms which are anticipated to increase in frequency and strength as the climate changes. Climate or CO_2_ induced changes in pollen development and morphology and the consequences for increased fragmentation and allergenicity remain an understudied aspect of plant based aero-allergens.

### Demographics

Allergenicity of pollen is a function of species. Ragweed, ryegrass, elm, oak all illicit different responses. But the distribution and pollen load of these species is in flux. Increasing CO_2_ and altered temperature and precipitation are likely to affect allergenic weed demography (19, 20), including establishment (21), competition (22), distribution (23), and management (24).

For example, there is strong evidence that ragweed, a widely recognized cause of allergic rhinitis in North America has benefitted from climate change, and is likely to expand its northern ranges, while contracting its southern populations (25). For woody perennial species expansion of Juniperus species in the United States has been noted over several decades, including potential links with both climate change and increasing CO_2_ concentrations (26). Overall, demographic flux needs to be considered in pollen monitoring. Yet in examining long-term records of pollen at the decadal level the list of allergenic species is often static and does not always reflect species that are new or diminishing in population (5).

### Vernalization

There are a number of studies that have shown a role of temperature and/or carbon dioxide concentration on advancing pollen release times for various allergenic tree species (20). However, the temporal role of rising temperatures and additional CO_2_ is also dependent on vernalization, the induction of flowering necessitated by exposure to cold (winter) temperatures. Warmer temperatures over winter could reduce floral initiation, but warmer spring temperatures could accelerate floral bud development and opening. While trees do release aeroallergens in the spring, warmer winters may result in earlier flowering, or flowering delays or even decreased floral numbers, depending on the tree species' specific need for vernalization (27). Hence, there can be both earlier and later allergen exposures from trees, depending on species, and location. Overall, changes in pollen season length, allergenicity and pollen amounts have not been well-quantified, particularly for hardwood trees with respect to rising CO_2_, or CO_2_ and temperature. Understanding vernalization in the context of climate change may be critical to assess current and future trends in tree pollen production.

## Indirect Plant/Environment Interactions

### Fungal Interactions

Although the function of climate in the induction and spread of molds is well-recognized (28), it is important to also emphasize the role of plants as hosts in fungal biology. Of particular interest in this regard is *Alternaria alternata*, an opportunistic fungal pathogen with hundreds of plant hosts, but whose spores are also recognized as a significant aeroallergen source associated with allergic dermatitis. It is interesting to note that rising levels of CO_2_ and/or climate change are likely to affect *Alternaria*'s plant hosts, including CO_2_ induced declines in the nitrogen content of plant tissue (29). As a result it is interesting to ask whether these changes can also alter *Alternaria* life cycle, with potential changes in sporulation.

One of the most interesting studies in this regard is by Wolf et al. (30) where the effects of rising CO_2_ concentration on the quantity and quality of *Alternaria* fungal spores was studied using timothy grass (*Phleum pratense*) leaves as the growth medium. Here the researchers found a shift in reproductive phenology where CO_2_-induced declines in leaf concentration resulted in a earlier shift for sporulation in *Alternaria*. Overall, at the higher CO_2_ concentrations (500 and 600 ppm), *A. alternata* produced nearly three times the number of spores and more than twice the total antigenic protein per plant relative to ambient CO_2_ conditions.

This is an indirect effect relative to plant biology *per se*; it illustrates the role of rising CO_2_ on qualitative changes in plant tissue, which in turn may have significant global interactions with respect to fungal sporulation. However, at present, the extent of the interaction between CO_2_ stimulation, changes in leaf tissue chemistry and fungal sporulation capacity has not been well-characterized.

### Air Quality and Allergenic Pollen

Air pollution has been associated with increased permeability and easier penetration of pollen allergens into airways of susceptible subjects (31). Exposure to ozone for example may increase sensitization to outdoor aeroallergens (32), including plant based pollen (33); and there are projections that climate change could increase near-surface ozone concentration in urban areas (34).

But there is also an additional plant biological aspect that is of interest. Plants release volatile organic compounds, or VOCs, into the air when attached by insects. These VOCs in turn are detected by other plants who can increase their production of VOCs to ward off herbivory. And it is those VOCs that are also a source of air pollution—a chemical determinant of ground level ozone.

**Figure 1:**
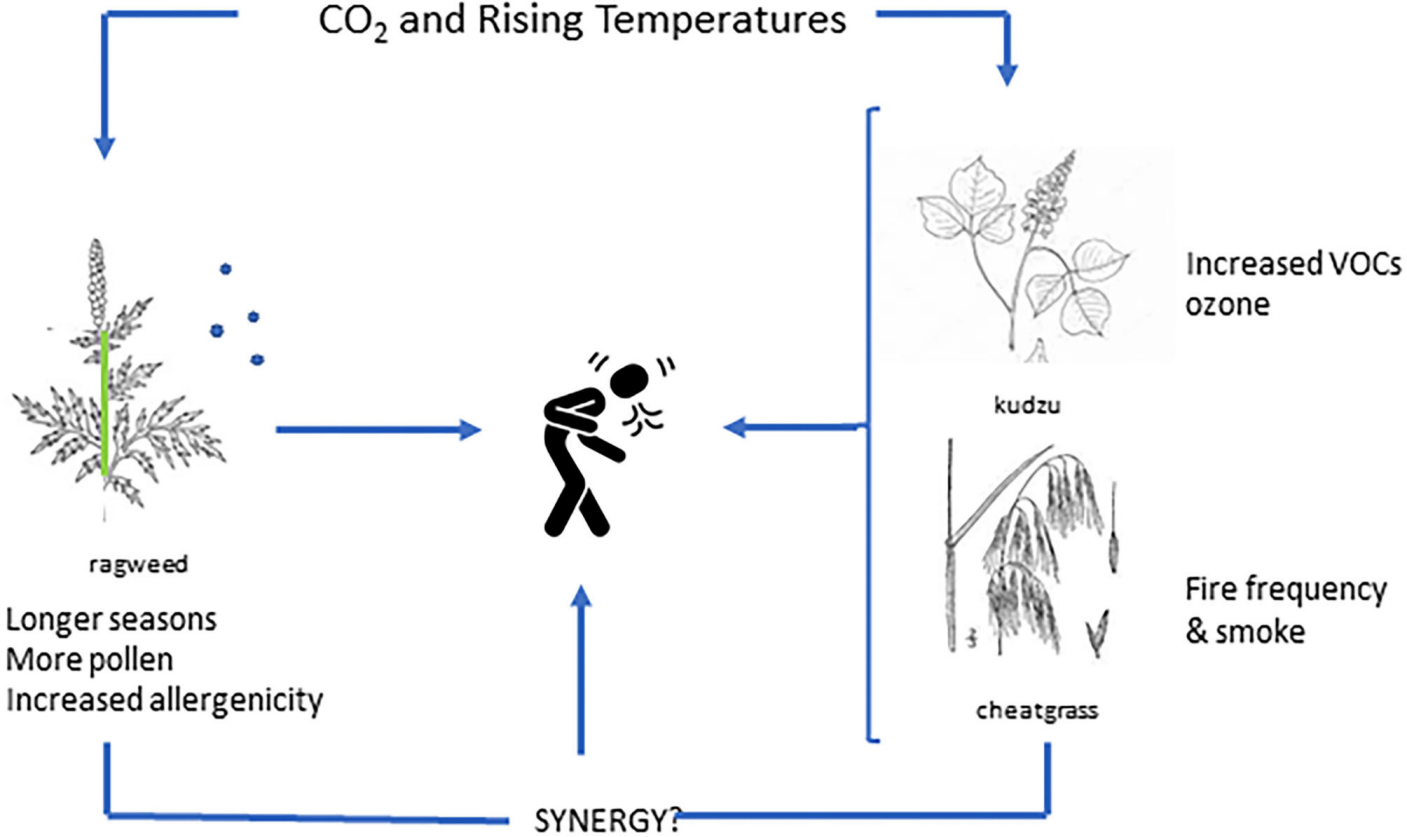
Illustration of individual and synergistic impacts related to plant based aeroallergens in the context of rising levels of carbon dioxide and/or temperature.

One of the most prominent plant sources of VOCs, specifically isoprene, is kudzu, an invasive vine found extensively throughout the southeastern U.S., but is also migrating northward (35). Kudzu is such a powerful VOC source that extensive kudzu invasion can lead to an increase in the number of high ozone events (above 70 ppb) of up to 7 days each summer for some regions (36). Rising CO_2_ has also been shown to stimulate kudzu growth (37, 38), but specific interactions with CO_2_ and temperature on isoprene release, ozone intensification and potential synergy with aero-allergens remain to be determined (39) (Figure 1).

This illustrates the complexity of botanical responses to rising CO_2_ and a warming climate; changes in VOCs could also increase ground level ozone, potentially exacerbating the impact of allergenic pollen in a public health context. Conversely, additional ozone could limit tree growth and pollen production, although, interestingly, supra-ambient ozone levels appear to have little effect on ragweed (40).

In addition to ozone, another air quality aspect that deserves additional consideration relative to allergic rhinitis is wildfire. Wildfire smoke contains a complex mixture of carcinogenic and respiratory substances including VOCs, ozone and particulate matter (PM). In addition to VOCs and ozone, there are a number of epidemiological studies confirming the association of PM with allergic respiratory diseases [e.g., (41)]. Particulate matter, in turn, is associated with fire, and fire acreage and intensity are changing in response to warming temperatures and shifting precipitation patterns (42). Interactions between climate induced changes in fire frequency / intensity and climate/CO_2_ increases in seasonal aeroallergens have not been extensively investigated (43).

Here too there is an additional botanical aspect. Plant material is the primary fuel for wildfires. As such, can rising CO_2_ levels and/or climate change alter plant tissue and combustibility, flammability or smoke quality? It can be argued that such changes would, in turn, also affect fire dynamics and allergenic sensitivity.

Cheatgrass (*Bromus tectorum*), is an invasive grass species found throughout much of Western North America. It can grow and thrive on little water, leaving dense continuous mats of flammable vegetation with subsequent increases in fire frequency. Interestingly, different cohorts of cheat-grass grown under atmospheric CO_2_ levels (from 270 to 420 ppm at 50 ppm intervals) suggested that recent increases in CO_2_ may have contributed to cheat-grass productivity and fuel load as well as increased combustibility (44). Additional information as to the role of CO_2_ and/or climate change on plant tissue composition related to fire dynamics are needed; however, interactions between CO_2_ and climate change with respect to wildfires, smoke characteristics (e.g., PM size) and seasonal allergen exposure are deserving of additional research to determine potential synergies (Figure 1).

### Pesticide Usage

Health studies directed to agricultural workers indicate a significant association between pesticide application and allergic rhinitis (45). Farm and ranch safety survey data from 11,210 farm operators indicated that 40% used pesticides, 30.8% had lifetime allergic rhinitis and ~5% currently suffered from asthma (46). Sensitivity may also vary by pesticide category; for example Slager et al. (46) noted that the herbicides glyphosate and petroleum oil were associated with current rhinitis and increased rhinitis episodes. Although additional data is needed to clarify the dose-response relationship between pesticide use and negative respiratory health effects, it is clear that insecticide and herbicide use are significantly associated with lifetime allergic rhinitis and existing asthma (47).

There is an additional plant biology aspect to this association. Decreases in the efficacy of different pesticides, including herbicides, has been detected under climatic changes associated with rising temperature and CO_2_ enrichment (48–50). Such declines may prompt increases in pesticide application (concentration and timing), already a concern due to high levels of selection pressure and increased resistance imposed by herbicides (the most frequently applied pesticide category).

The effect of rising CO_2_ and temperature on pests, particularly weeds, may result in additional pesticide applications, exacerbating responses related to plant aero-allergen production. However, to date, there are no studies that have considered their potential interaction.

## Unknowns and Challenges

Increasing levels of CO_2_ and rising temperatures are a ubiquitous aspect of global climate change. The extent of climatic change and the associated uncertainty are anticipated to have wide-spread negative impacts on human systems (51), threatening to negate years of progress in public health. Such impacts focus, understandably, on direct physical consequences on human health, such as hurricanes, floods, fires; sudden environmental shifts related to hypothermia or cardiovascular function; biological consequences can include increased risk of vector borne diseases such as malaria, dengue fever and west Nile virus (52).

And there are botanical consequences, aside from crop systems and food availability, that are also deserving of attention. In this regard there is increasing consensus that plant-based aeroallergens are likely to be significantly altered directly by rising CO_2_ and/or climate, with earlier pollen initiation, greater pollen loads, greater allergenicity, and longer seasonal exposure.

Although there is a merited focus on negative affects with respect to pollen and allergenicity, other effects which could reduce pollen spatially or temporally also deserve consideration. For example, climate change may limit suitable habitat for known allergenic tree species, such as birch (53). Warming winter temperatures may also delay anthesis in winter-flowering species or alter the vernalization requirement for other tree species (54). Temperature influences on masting in oak, or other climatological influences on temporal flowering could, potentially, affect pollen seasonality or pollen load (20), with potential differences between perennial and herbaceous species (55).

To fully understand health consequences related to aero-allergens requires a more fundamental appreciation of the complexity of plants and plant systems. Climate and CO_2_ will directly alter core aspects of plant development, from height to vernalization to their geographic distribution, all characteristics that will modify pollen production, allergenicity and airborne retention times, but remain, overall, overlooked with respect to aero-allergen impacts. Similarly, there are biological interactions that deserve additional scrutiny, from CO_2_ induced changes in plant chemistry and subsequent effects on fungal aero-allergen production; to CO_2_ induced increases in VOCs, and potential synergy between ozone and aero-allergens; to climate/CO_2_ changes in plant flammability that could alter smoke characteristics and aero-allergen sensitivity. These examples are of interest but are not the only potential interactions that could occur. For example, there is very preliminary information that rising CO_2_ could increase allergen content in some peanut lines (56); if food allergies are in fact, altered by CO_2_/climate, what are the ramifications for other allergies, including aeroallergen, sensitivity?

The occurrence of allergic rhinitis globally is estimated at around 500 million (57). A recent seminal review by Beggs (58) indicates that the increase in allergic rhinitis and associated asthma and aeroallergens is increasing significantly, and it is clear that the research and collaborations that have been conducted to examine climate and CO_2_ have been critical in establishing the botanical links to spatial and temporal trends in aero-allergens.

But it is also clear that there is more to discover. The research topics that have been illustrated here are important, but they are reflective of my own botanical bias. Other work, from understanding the role of climate change to indoor and outdoor allergen trends and interactions, to urban heat island macro-environments, to synergism of aeroallergens with other environmental triggers, to clinical responses that document respiratory health effects, are also essential, critical. Overall, in examining the role of climate change on public health as it pertains to plant based aeroallergens, there is both recognition of what has been accomplished, and how many challenges still remain.

## Author Contributions

The author confirms being the sole contributor of this work and has approved it for publication.

## Conflict of Interest

The author declares that the research was conducted in the absence of any commercial or financial relationships that could be construed as a potential conflict of interest.

## Publisher's Note

All claims expressed in this article are solely those of the authors and do not necessarily represent those of their affiliated organizations, or those of the publisher, the editors and the reviewers. Any product that may be evaluated in this article, or claim that may be made by its manufacturer, is not guaranteed or endorsed by the publisher.
